# An evaluation of subtalar titanium screw arthroereisis for the treatment of symptomatic paediatric flatfeet - early results

**DOI:** 10.1186/s12891-023-06937-2

**Published:** 2023-10-19

**Authors:** Anna Szesz, Krzysztof Małecki, Marcin Sibiński, Kryspin R. Niedzielski

**Affiliations:** 1 Clinic of Orthopaedics and Traumatology, Polish Mother’s Hospital Research Institute, Lodz, Poland; 2 Clinic of Orthopaedics and Paediatric Orthopaedics, Medical University of Lodz, ul Pomorska 251, Lodz, 92-213 Poland

**Keywords:** Extraarticular sutalar implant, Flatfeet, Footprint, Pedography, Radiological measurements, Sinus tarsi implant

## Abstract

**Background:**

Idiopathic flexible flatfoot is a common condition in children which typically improves with age and remains asymptomatic. However, the condition can sometimes be more severe, and cause mechanical impairment or pain. The aim of the study was to perform a prospective clinical, radiological, podoscopic and pedobarographic assessment (static and dynamic) of subtalar titanium screw arthroereisis for the treatment of symptomatic, idiopathic, flexible flatfeet.

**Methods:**

A prospective, consecutive, non-controlled, cohort, clinical follow-up study was performed. In total, 30 patients (41 feet), mean age 10 (6 to 16 years), were evaluated. Clinical and standing radiological assessments, static and dynamic pedobarography, as well as podoscopy, were performed before surgery and at final follow-up.

**Results:**

Treatment was associated with significant improvements in heel valgus angle, radiographic parameters (lateral and dorso-planar talo-first metatarsal angle, calcaneal inclination angle, talar declination angle, longitudinal arch angle) and podoscopic parameters (Clark’s angle, Staheli’s arch index and Chippaux-Smirak index). Significant increases were noted for lateral loading, forefoot contact phase and double support / swing phase, and reduced medial loading (dynamic pedobarography), as well as lateral midfoot area and loading, but decreased were observed for medial forefoot loading (static pedobarography). Four patients reported persistent pain in the sinus tarsi region (six feet), and in one case, the implant was replaced for a larger one due to undercorrection. No overcorrections or infection complications were noted in the study group.

**Conclusions:**

Subtalar arthroereisis is a minimally-invasive and effective surgical method for treating symptomatic, idiopathic, flexible flatfeet; it has an acceptable complication rate with good early clinical results.

**Level of evidence:**

II b.

## Highlights

Subtalar artroereisis is an effective surgical technique for correcting flexible flatfeet.

It offers minimal invasiveness, acceptable complication rate and high patient satisfaction after surgery, even if undercorrection is present.

## Background

Flexible flatfoot is a common condition in the paediatric population and one of the most common causes of consultation with a paediatric orthopaedist. In a child, the longitudinal arch of the foot is initially flat, and then gradually rises to reach its target height at about eight to ten years of age [1–3]. In the vast majority of cases, static flatfeet is asymptomatic; however, due to the lack of specific common standards regarding the height of the longitudinal arch, it is difficult to determine the true incidence in children [2]. In an epidemiological study by Pfeiffer et al. based on 800 children aged three to six years [2], was identified in 54% of children aged three years. This value fell to 29% at six years of age without treatment. Harris and Beath [4] report physiological flatfeet in 23% of adults, while a Polish study of 3600 children found the incidence of flatfoot together with valgus hindfoot to be 12.73% [5].

Despite the widespread use of insoles in children, their effectiveness in treating asymptomatic flatfeet is controversial, as is the use of rehabilitation exercises [1, 3, 6]. It is unclear whether the effect of these insoles may depend on the physiological development of the foot; however, while the corrective effect of the insoles remains uncertain, they may still relieve pain [1, 6, 7]. Therefore, it is recommended that symptomatic static flatfeet should first be treated conservatively using different types of custom-moulded or prefabricated orthoses. Surgical treatment is indicated only when conservative treatment is ineffective due to persistent pain [1, 6, 7].

Other techniques for correcting flatfeet include calcaneal osteotomy, with the most popular being medial sliding osteotomy, sliding-closing medial calcaneal osteotomy or lateral column lengthening calcaneal osteotomy by Evans [8, 9]. Alternatively, subtalar arthroeresis can be used, although it results in in partial limitation of mobility in the talocalcaneal joint due to the introduction of various types of implants. Over the years, various implants have been used, ranging from bone blocks and silicone implants, to bioabsorbable and metal screws screwed into the calcaneus (calcaneo-stop technique); however, modern procedures are based on titanium implants, which are inserted into the sinus tarsi, sometimes up to the tarsal canal [10].

Currently, the most popular surgical techniques for correcting flatfeet are arthroeresis and calcaneal lengthening [9, 11]. Both techniques provide a significant improvement in radiological parameters, and neither has been found to offer significant advantages over the other [12]. However, in our opinion, arthroeresis is superior to calcaneal osteotomy, due to it being minimally invasive, easier to perform and more willingly accepted by children and their parents, and the fact that it allows a faster return to full activity. In addition, in the case of complaints or insufficient correction, the procedure can also be reversed by removing or replacing the implant.

However, there is a need for a more conclusive evaluation of the effectiveness of subtalar titanium screw arthroereisis. Therefore, the aim of the present study was to perform a prospective clinical, radiological, podoscopic and pedobarographic assessment (static and dynamic) of subtalar titanium screw arthroereisis for the treatment of symptomatic, idiopathic, flexible flatfoot.

Almost all authors concentrate on objectives aspects of the procedure [1, 13–16]. The study itself is based on a wide spectrum of objective clinical, radiological, podoscopic and pedobarographic measurements, together with subjective observations based on a specifically-designed foot health survey completed by the patients.

## Methods

A prospective consecutive, non-controlled, clinical follow-up study was performed. The cohort comprised 32 patients with symptomatic flexible flatfoot, after failure of conservative treatment; all had been treated with subtalar arthroereisis from February 2017 to December 2018 in the hospital of the first author. The exclusion criteria comprised treatment of *pes eqiunovarus*, the presence of genetic or neurological disorders, previous foot injury or surgical intervention on the foot, or the inability to follow directions during gait examination.

Two patients were lost to follow up and discharged from the study; therefore, 30 patients (41 feet) were included in the final assessment. Mean age was 10 years (6 to 16 years). Assessment was performed before surgery and after a mean period of eight months (6 to 12 months).

The final follow-up was based on a simple patient foot health survey prepared for the study. It included the following areas: pain in the operated foot during the previous week (viz. no pain, mild, moderate, severe), pain during activities (viz. no pain, at every day activities, long walking, sport activities, walking on uneven surface, walking on stairs) and well-being in context of the operated foot (viz. very good, good, moderate, poor).

The initial assessment included a clinical examination comprising the following indications for surgery: flexibility of deformation, degree of heel valgus, presence of laxity, symptoms and Achilles tendon contracture.

Heel valgus was assessed before and after surgery by the patient standing backwards on a treadmill. A photo was taken with a camera placed on a tripod 40 cm above the ground. In both cases, the angle of valgus of the heel was determined using a protractor in the treadmill gait analysis software (Freestep, Sensor Medica, Rome, Italy).

Tissue flaccidity was assessed using the Beighton scale. A Beighton score of 0–4 was assumed as indicating normal values, while 5–9 indicated tissue flaccidity [17].

Dorsoplanar and lateral radiographs were obtained during full weightbearing in a standing position. The radiographs were measured digitally with CGM DiagRAD software (CompuGroup Medical, Lublin, Poland). Radiographic measurements were obtained at lateral and dorsoplanar talo-1st metatarsal angle (TMT 1), calcaneal inclination angle, talar declination angle and longitudinal arch angle, as described by Davids et al. [18, 19].

Static and dynamic pedobarography was performed on a treadmill with a diagnostic platform (Run time treadmill, Sensor Medica, Rome, Italy) and Freestep software (Sensor Medica, Rome, Italy).

During dynamic pedobarography, the patient was instructed to walk on the treadmill. Whole stance time, double support/swing phase, stance phase time, initial contact phase (ICP), forefoot contact phase (FFCP), flat foot phase (FFP), and foot contact area were measured. In addition, the percentage loading of the forefoot, hindfoot, medial and lateral parts of the foot was evaluated while in the flatfoot phase.

During static pedobarography, each subject was instructed to stand on a pressure sensing device with their body weight distributed equally over both feet. The feet were subjected to digital mapping during static examination; this included the lateral and medial forefoot, lateral and medial midfoot and lateral and medial hindfoot.

**Fig. 1:**
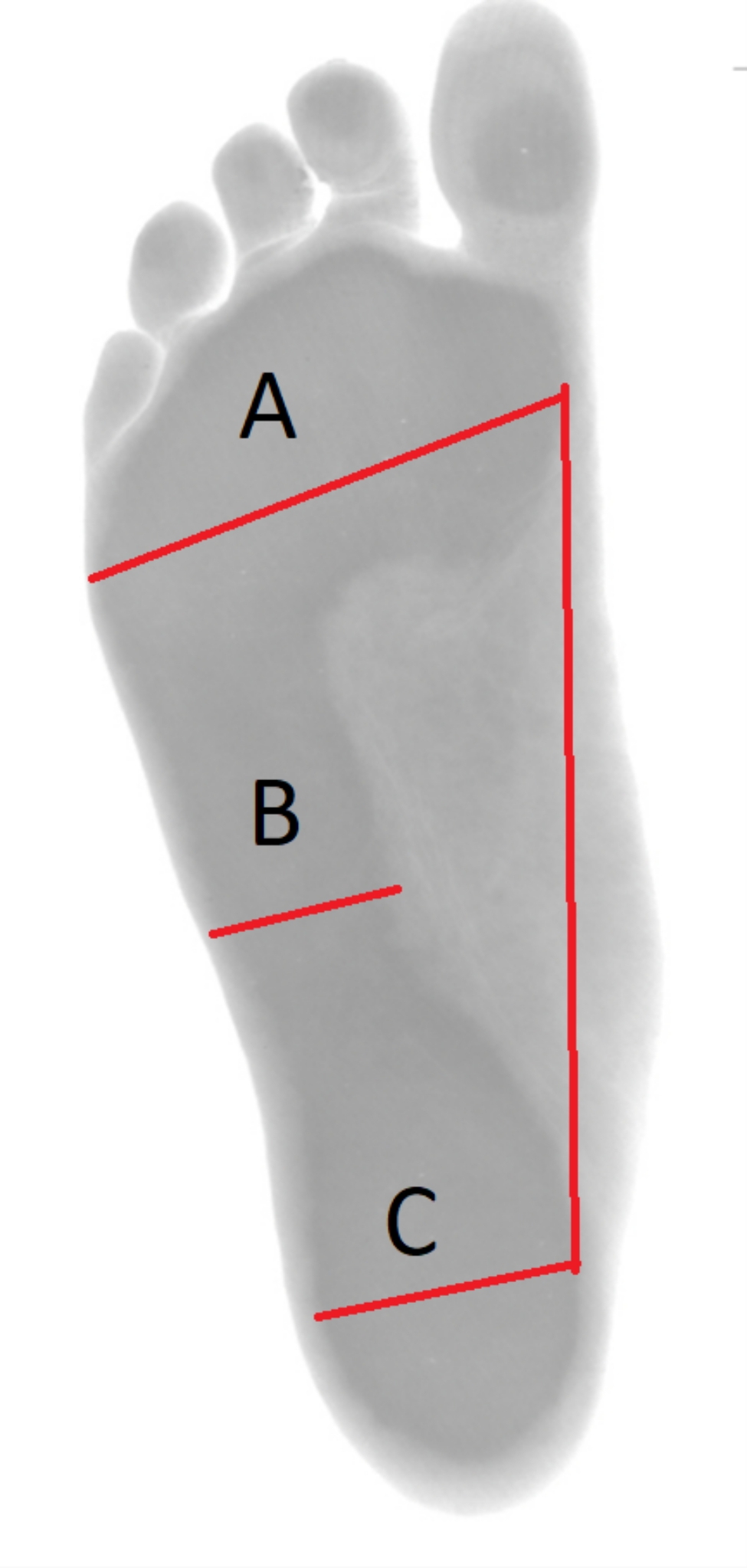
The method of measuring Staheli’s arch index (B/C ratio) and Chippaux-Smirak index (A/B*100% ratio)

**Fig. 2:**
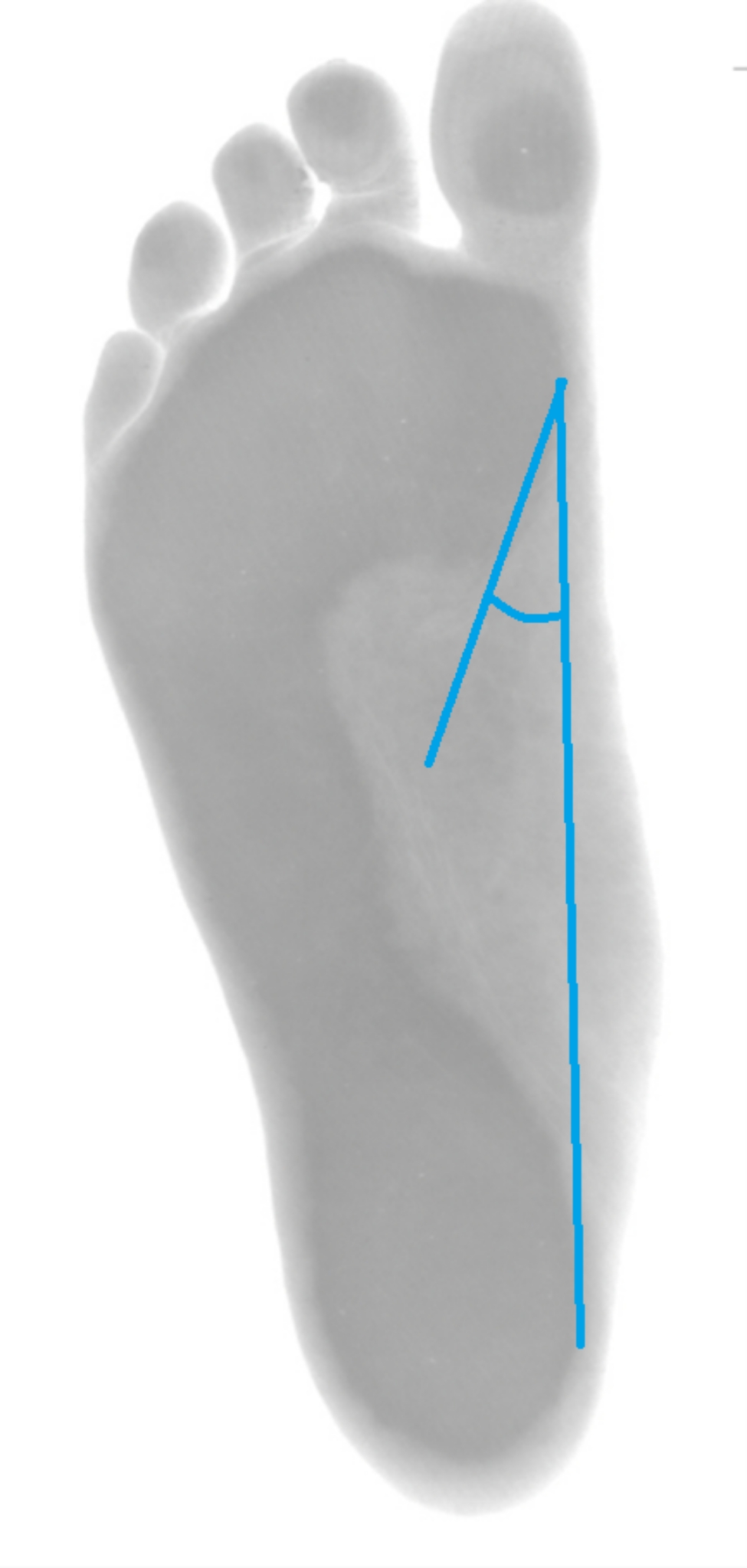
The method of measuring Clarke’s angle during podoscopy

The footprints were recorded using a footprint digital scanner (Sensor Medica, Rome, Italy) using Freestep software (Sensor Medica, Rome, Italy). The flatness of the footprint was measured by measuring, Staheli’s arch index (SAI), Chippaux-Smirak index (Fig. 1) and Clark’s angle (CSI) (Fig. 2) [1, 19].

The B/C ratio was used to determine SAI: a value of 0.44–0.89 indicated a normal rate, < 0.44 as hollow foot and > 0.89 as flat foot [1, 19]. The A/B*100% ratio was used to calculate CSI; a value of 26–45% indicated a normal value, 46–49% flat foot I°, 50–75% flat foot II°, > 75% extreme flat foot and ≤ 25% hollow foot [1, 19].

Surgical technique.

**Fig. 3:**
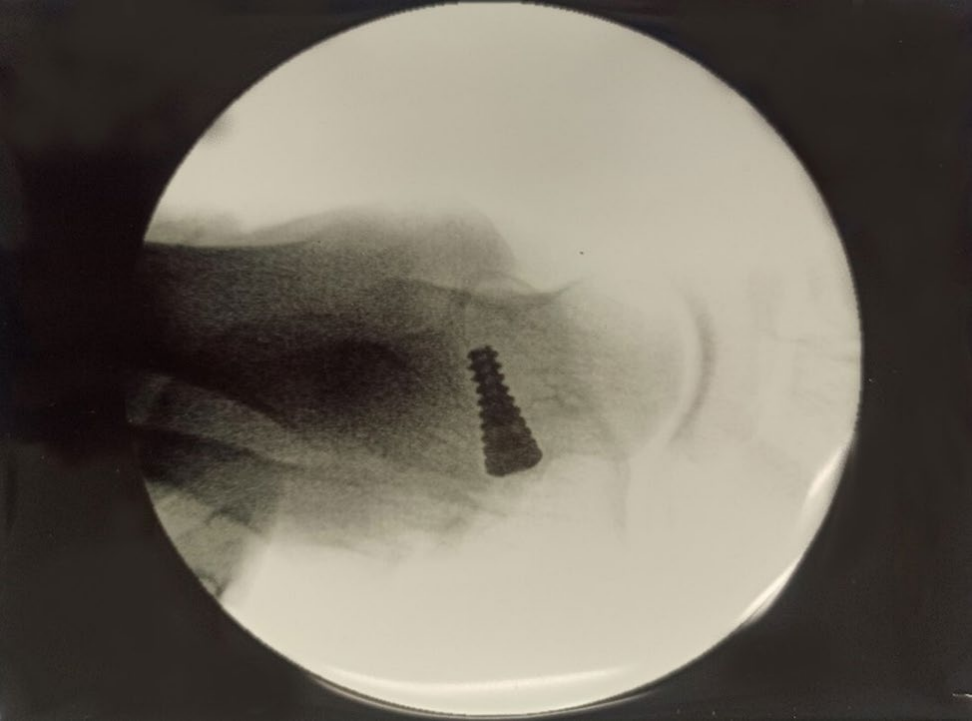
Intraoperative fluoroscopy after subtalar arthroereisis – AP view

**Fig. 4:**
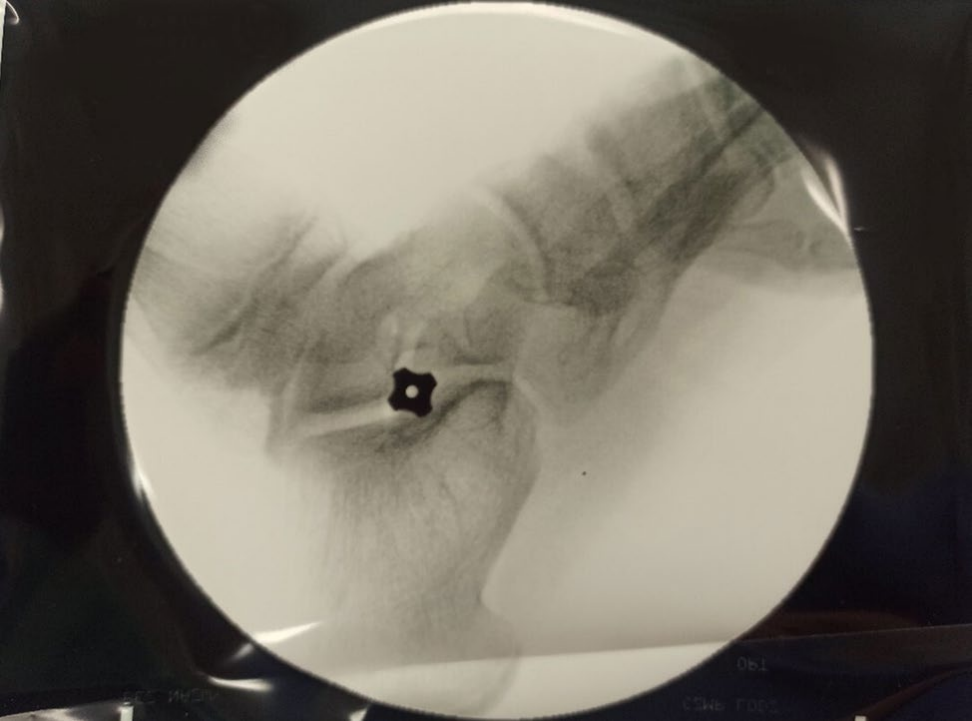
Intraoperative fluoroscopy after subtalar arthroereisis – lateral view

A skin incision measuring approximately 1–2 cm was made over the sinus tarsi. A blunt dissection was then made through the superficial tissues and further to the sinus tarsi to spread apart the interosseus ligament. A blunt guide wire was then placed in the sinus and canalis tarsi, so that the end of the wire was felt below the medial malleolus. The correct size of the implant was determined using trial sizers; the position of the guide wire and sizers were checked with fluoroscopic imaging. Following this, the movements of the foot were examined, with the goal being to achieve up to 5° of pronation. Care was taken especially to avoid overcorrection. The exact size of the implant was established based on x-ray imaging and clinical examination, the implant was placed and the wound was closed. A titanium conical screw implant was used (Figs. 3 and 4).

The study was approved by the Bioethical Committee of the institution of the first author (project number 2017/IV/62-MN). It was performed in line with the principles of the Declaration of Helsinki. Informed consent was obtained from the parents and/or legal guardians of the participants included in the study. If the patients were 13 years old or above, informed consent was obtained from both the participants and their parent and/or legal guardians, as required by Polish law.

### Statistical analysis

The following descriptive statistics were created for the measured values: weighted arithmetic mean, standard deviation, 95% confidence interval and minimum-to-maximum values. All data was first tested for normality, skewness and kurtosis, and the equality of variance was confirmed using Levene’s test.

For normally-distributed variables, the differences in the values before and after surgery were analysed using multifactor analysis of variance (ANOVA) with repeated measurements. For non-normally distributed variables, generalized estimating equations (GEE) with repeated measurements and robust standard errors (i.e. sandwich estimators) were fitted. All the statistical models were controlled for sex. As the measurement unit was a single foot, and considering that some participants underwent surgery on one foot and others on both feet, an intra-subject correction was applied. Any comparisons with P < 0.05 were deemed statistically significant. All statistical analyses were carried out using Stata/Special Edition, release 14.2 (StataCorp LP, College Station, Texas, USA). Patients lost to follow-up were not included in the statistical analysis.

## Results

The final follow-up, i.e. after a mean period of eight months (6 to 12 months) included 30 patients (41 feet). During this follow-up, 26 patients reported no pain in the operated foot during the last week, two reported mild pain, and two moderate pain. In addition, 26 patients reported no pain during activities, two during everyday activities, two after long walks, four during sport activities, two during walking on uneven surface and one during walking on stairs. The level of well-being regarding the operated foot was assessed as very good by 20, good by six, moderate by two and poor by two; no other complications were reported in the remaining patients. All patients assessed their general well-being with regard to the foot as very good.

**Fig. 5:**
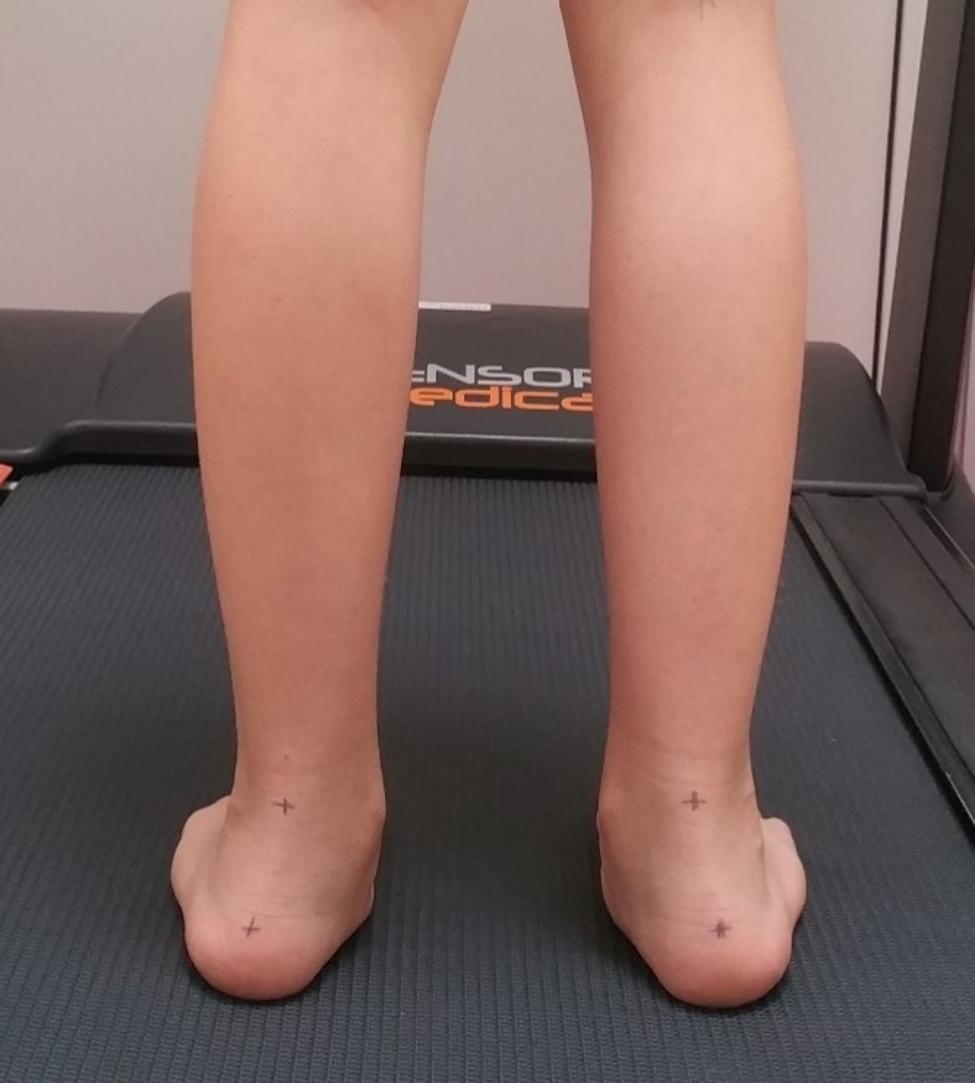
Valgus deformity of the hindfoot before surgery

**Fig. 6:**
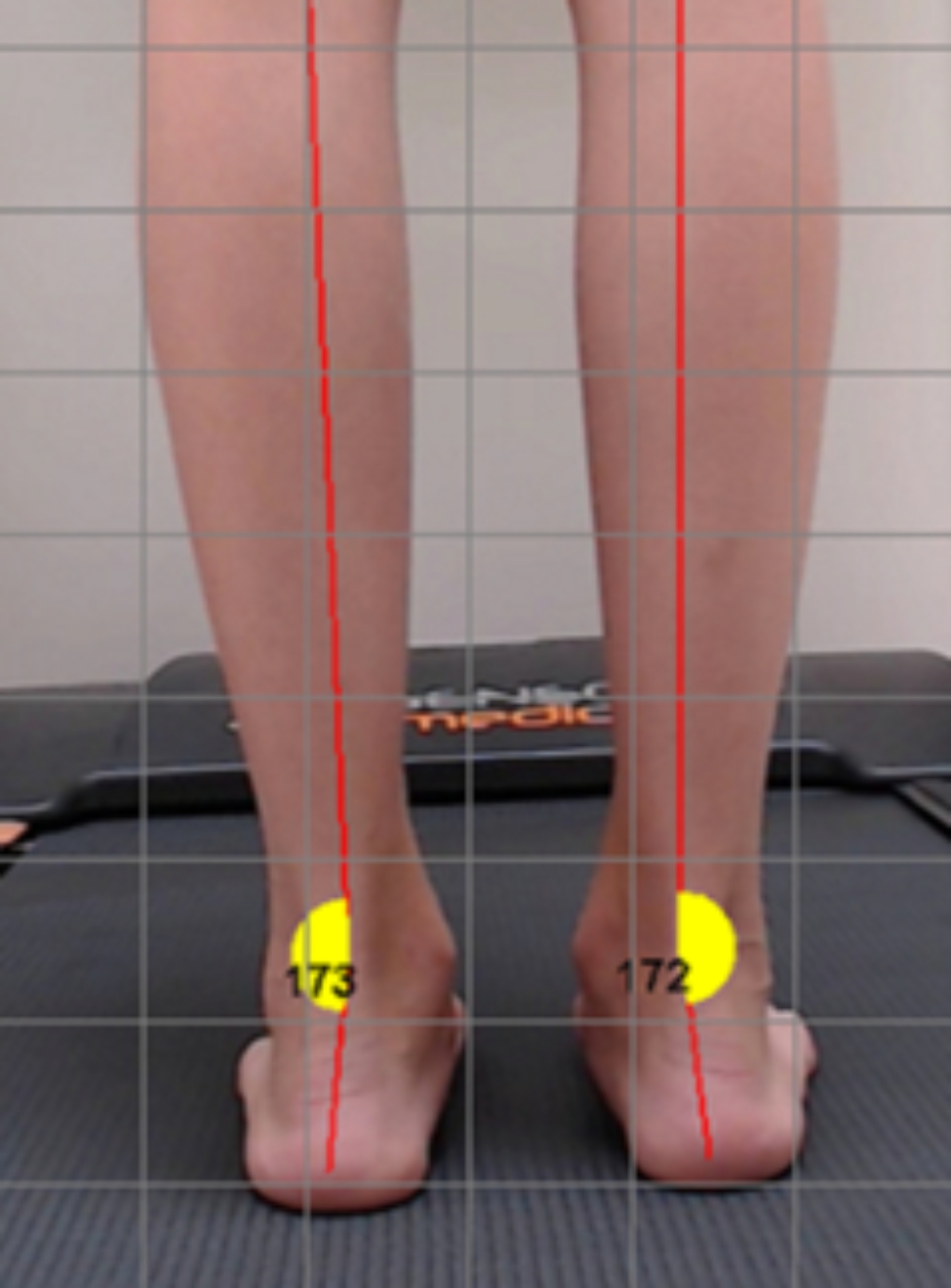
Valgus deformity after surgical correction at last follow-up

The mean level of correction of heel valgus after surgery was 5.4° (range from 8° to 25°). Satisfactory correction (tarsal valgus < 10°) was achieved in 83% of patients. Tarsal valgus exceeded 10° after surgery in 13% of patients (Figs. 5 and 6) (Table 1). No overcorrection of heel valgus was noted in any of the patients.

**Table 1:** Descriptive statistics for the data from the heel valgus and X-ray images before surgery and at last follow-up

Parameter	Phase of the study	Statistical parameter					p-value
		M*	Me^†^	SD^‡^	95% CI**	Min. – max.	
Heel valgus angle	Before surgery	13.7°	13.5°	3.9°	12.4°-14.9°	8°-25°	**< 0.001**
	last follow-up	8.1°	8.0°	2.4°	7.3°-8.9°	3°-14°	
Talo-metatarsal angle I (lateral view)	Before surgery	21.2°	22.0°	6.7°	19.0°-23.3°	8°-37°	**< 0.001**
	last follow-up	8.6°	9.0°	6.4°	6.6°-10.6°	0°-24°	
Talo-metatarsal angle I AP (dorso-planar view)	Before surgery	18.4°	17.5°	7.8°	15.9°-20.9°	7°-39°	**< 0.001**
	last follow-up	7.1°	5.0°	7.3°	4.8°-9.5°	0°-30°	
Calcaneal inclination angle (calcaneal pitch)	Before surgery	11.5°	10.0°	4.7°	9.9°-13.0°	5°-25°	**< 0.001**
	last follow-up	14.3°	13.5°	4.8°	12.8°-15.9°	6°-27°	
Talar declination angle	Before surgery	37.3°	37.0°	4.9°	35.7°-38.8°	28°-52°	**< 0.001**
	last follow-up	26. °	26.0°	5.1°	24.4°-27.6°	12°-39°	
Longitudinal arch angle	Before surgery	163.4°	164.0°	7.5°	160.7°-165.8°	146°-180°	**< 0.001**
	last follow-up	162.3°	162.0°	6.2°	160.3°-164.3°	146°-180°	

(* M – mean; ^*†*^ *Me – median;* ^*‡*^ SD – standard deviation; ** CI – confidence interval.) Values marked in bold are statistically significant

No infection complications were reported, and any minor ailments around the operated site receded within three months of the procedure. One patient had the implant replaced with a larger one due to unsatisfactory correction; after replacement, the correction was satisfactory and there was no pain. Hence, the total incidence of complications was 16%. In addition, four of the 30 enrolled patients scored five points or more on the Beighton scale, indicating tissue flaccidity (13% of the study group). Radiological analysis indicated a statistically significant (p < 0.001) change in all tested parameters after the procedure; however, not all patients achieved normative values for a given measurement. The greatest change was observed for the TMT 1 angle on dorso-planar and lateral view, and the talar declination angle (Table 1).

At the last follow-up, dynamic pedobarography revealed a significant prolongation of double support/swing and FFCP. In addition, a significant increase was also noted in the load on the lateral edge of the foot, as well as reduced medial loading (Table 2). Static pedobarography identified a statistically significant increase in loading in the lateral midfoot area, and a decrease in medial forefoot loading (Table 3). The podoscope examination revealed a significant improvement in all examined parameters (Table 4).

**Table 2:** Descriptive statistics for the dynamic pedobarography results before surgery and at last follow-up

Investigated trait	Phase of the study	Statistical parameter					p-value
		M	Me	SD	95% CI	Min. – max.	
Stance time (msec)	Before	953.3	943.0	179.1	896.0-1010.5	678–1467	0.5
	After	973.7	957.0	118.4	935.8-1011.6	774–1272	
Double support / swing (msec)	Before	241.0	235.0	59.7	221.9-260.1	124–367	**0.005**
	After	278.9	263.5	65.5	257.9-299.9	189–525	
Stance phase time (msec)	Before	592.6	561.0	165.3	539.8-645.5	345–1031	0.9
	After	600.5	557.5	186.5	540.9-660.2	434–1356	
Initial contact phase. ICP (msec)	Before	115.1	97.0	80.0	89.5-140.6	6-321	0.2
	After	94.3	93.5	35.7	82.8-105.7	22–169	
Forefoot contact phase. FFCP (msec)	Before	571.5	569.0	117.2	534.0-608.9	300–854	**< 0.001**
	After	654.7	662.5	86.0	627.2-682.2	411–839	
Flat foot phase. FFP (msec)	Before	239.0	219.5	117.9	201.3-276.7	95–616	0.124
	After	209.5	189.0	82.3	183.2-235.8	73–441	
Foot contact area (cm^2^)	Before surgery	163.8	161.5	34.6	152.8-174.9	109–268	0. 3
	last follow-up	158.7	155.5	24.7	150.9-166.6	112–236	
Forefoot loading (%)	Before surgery	72.9	72.0	8.9	70.0-75.7	54–89	0.7
	last follow-up	72.3	71.50	6.5	70.2–74.4	60–86	
Hindfoot loading (%)	Before surgery	27.1	28.0	8.9	24.3–30.0	11–46	0.7
	last follow-up	27.7	28.5	6.5	25.7–29.8	14–40	
Medial loading (%)	Before surgery	51.6	52.0	6.4	49.6–53.7	39–64	**< 0.001**
	last follow-up	43.5	43.0	6.6	41.4–45.6	33–56	
Lateral loading (%)	Before surgery	48.4	48.0	6.4	46.3–50.4	36–61	**< 0.001**
	last follow-up	56.5	57.0	6.6	54.4–58.6	44–67	

(* M – mean; ^*†*^ *Me – median;* ^*‡*^ SD – standard deviation; ** CI – confidence interval.) Values marked in bold are statistically significant

**Table 3:** Descriptive statistics for the static analyses before surgery and at last follow-up, with statistical analysis

Investigated trait	Phase of the study	Statistical parameter					p-value
		M	Me	SD	95% CI	Min. – max.	
Lateral forefoot area (cm^2^)	Before surgery	13.5	14.0	8.1	10.9–16.1	1–36	0.3
	last follow-up	14.5	14.0	5.8	12.6–16.3	4–30	
Lateral forefoot loading (%)	Before surgery	7.5	7.5	4.6	6.03-9.0	0–17	0.6
	last follow-up	7.8	7.0	4.1	6.5–9.1	1–18	
Medial forefoot area (cm^2^)	Before surgery	12.8	12.0	6.7	10.6–14.9	1–30	0.3
	last follow-up	11.8	10.5	6.0	9.8–13.7	0–26	
Medial forefoot loading (%)	Before surgery	7.5	7.0	4.3	6.1–8.8	1–17	**0.03**
	last follow-up	6.0	5.0	3.7	4.8–7.2	0–17	
Lateral midfoot area (cm^2^)	Before surgery	6.5	4.0	5.6	4.7–8.2	0–19	**< 0.001**
	last follow-up	10.0	10.0	4.4	8.6–11.4	0–22	
Lateral midfoot loading (%)	Before surgery	4.1	3.0	3.7	3.0-5.3	0–12	**0.004**
	last follow-up	5.7	6.0	3.2	4.6–6.7	0–13	
Medial midfoot area (cm^2^)	Before surgery	5.4	4.0	4.7	3.9–6.9	0–16	0.6
	last follow-up	5.0	5.0	3.3	4.0-6.1	0–14	
Medial midfoot loading (%)	Before surgery	3. 7	2.5	3.3	2.6–4.7	0–13	0.1
	last follow-up	2.9	2.0	2.4	2.2–3.7	0–10	
Lateral hindfoot area (cm^2^)	Before surgery	12.3	12.0	2.8	11.4–13.2	6–18	0.4
	last follow-up	12.6	12.0	2.5	11.8–13.4	9–19	
Lateral hindfoot loading (%)	Before surgery	11.22	10.50	3.57	10.1-12.37	5–20	0.4
	last follow-up	11.72	11.00	3.57	10.6–12.9	5–20	
Medial hindfoot area (cm^2^)	Before surgery	13.9	14.0	4.0	12.6–15.2	7–27	0.1
	last follow-up	13.1	13.0	3.0	12.1–14.0	8–24	
Medial hindfoot loading (%)	Before surgery	13.9	14.0	3.5	12.8–15.0	5–23	0.2
	last follow-up	13.1	14.0	3.2	12.0-14.1	7–24	
Total area (cm^2^)	Before surgery	68.9	67.0	20.1	62.5–75.4	36–110	0.3
	last follow-up	71.9	68.0	17.2	66.4–77.4	44–132	

(* M – mean; ^*†*^ *Me – median;* ^*‡*^ SD – standard deviation; ** CI – confidence interval.) Values marked in bold are statistically significant

**Table 4:** Descriptive statistics for the podoscopy outcomes before surgery and at the last follow-up, with statistical analysis

Angle / Index	Phase of the study	Statistical parameter					Level of statistical significance (p-value)
		M	Me	SD	95% CI	Min. – max.	
Clarke’s angle (deg)	Before surgery	31.0	31.0	14.4	26.4–35.6	3–55	**0.002**
	last follow-up	42.8	44.0	12.5	38.8–46.8	14–64	
Staheli index (SAI)	Before surgery	0.8	0.8	0.4	0.7-1.0	0.0-1.6	**0.003**
	last follow-up	0.6	0.6	0.2	0.6–0.7	0.3–1.2	
Chippaux-Smirak index	Before surgery	0.5	0.5	0.3	0.4–0.6	0.0-0.9	**0.002**
	last follow-up	0.4	0.3	0.1	0.3–0.4	0.2–0.8	

(* M – mean; ^*†*^ *Me – median;* ^*‡*^ SD – standard deviation; ** CI – confidence interval.) Values marked in bold are statistically significant

## Discussion

Our findings confirm that arthroeresis primarily corrects flatfoot by rectifying the subluxation in the ankle joint. This was expressed in our radiological images by reductions in talar declination angle, longitudinal arch angle and TMT 1 angle on lateral and dorso-planar view, together with an increase in valgus angle. The pedobarography data found correction to be achieved primarily by the load on the lateral edge of the foot being increased as a result of decreasing valgus foot deformity. Clinically, a reduction of heel valgus deformity was also observed, although the heel position was found to be corrected to a lesser degree than intended in 13% of patients.

Our findings are similar to those of previous studies, indicating that arthroeresis yields improved gait pattern and less pain [13–15]. Indino et al. found that subtalar arthroereisis with endorthesis was effective for treating paediatric flexible flatfoot, reflected in improved radiographic parameters at skeletal maturity [20]. In a study with a large number of patients, De Pellegrin and Moharamzadeh report a lower complications rate amongst those receiving subtalar arthroereisis with endorthesis compared to subtalar arthroereisis with calcaneo-stop [21]. In addition, the frequency of complications described in the present study is similar to that reported previously for subtalar titanium implants [13, 16, 21].

However, arthroeresis has its shortcomings. Firstly, it does not allow the size of the implant to be planned before surgery, and secondly, despite the implant being introduced under x-ray control, it only offers limited intraoperative assessment: the final result of the procedure is visible only during postoperative examination with the patient standing with a full load on the operated limb. The choice of implant size is crucial for a good postoperative result: a small implant will cause insufficient correction, and an oversized one will cause pain as a result of overcorrection [22].

Previous studies have found the frequency of complications after lower ankle arthroeresis to vary considerably [6, 15, 17]. The most common complaint is pain in the tarsal sinus area; however, it usually disappears after removal of the implant and most likely results from the use of an oversized implant [15]. No such complication was observed in our group of patients. Studies have reported some complications associated with implantation, with the most common deriving from its displacement and damage [6, 15, 17]. In some cases, with silicone and bioabsorbable implants, soft tissues can react to the implant material [13]. There are also isolated reports of talar neck fracture, talus osteonecrosis (when the STA-Peg implant is used), bilateral formation of talar cysts, or extensive synovitis in response to implant material [23–26]. However, these are isolated cases; by far the most common complaint is pain in the operated area associated with walking or prolonged effort. In our study group, four out of 30 patients reported problems persisting for more than six months after surgery.

A radiological study by Bourdet et al. [27] described four types of flatfeet, depending on the deformation found: (1) feet with a subtalar displacement (subtalar flatfoot), (2) feet with a deformity located mainly in the midfoot, with marked forefoot adduction and the top of the deformity at the height of the scaphoid and cubic bones, (3) feet with mixed subtalar-midfoot deformity (most common) and (4) flat-hollow feet with a lowering of the medial longitudinal arch, but a hollowing-out of the lateral arch, as expressed by an increased calcaneal-metatarsal V angle [27].

One possible reason for incomplete correction being observed in some patients, could be that while the participants were qualified for arthroeresis in our clinic due to the occurrence of pain in the course of static flat-valgus feet, the subtype of deformity was not specified. Interestingly, the best effects were obtained in patients in whom the deformity was expressed primarily as a subtalar subluxation; this was also reported in a previous study of surgical treatment of flat-valgus feet in adults using a titanium implant for the sinus tarsi and tarsal canal [28].

The pain accompanying in flatfoot may be associated with the greater work demanded of the short muscles of the foot due to the lack of “blockage” of the hindfoot. During walking, the foot should “lock” to form a rigid support to allow the foot to break out dynamically [29]. In the normal foot, the courses of the talonavicular and calcaneocuboid joint axes change with the heel position: while they run parallel when the heel is in the valgus position, thus allowing movement, these axes diverge as the position of the heel changes towards the varus, resulting in the centre of gravity shifting and the shin rotating outward. This change causes the movement to be blocked, the tarsus stiffening and forming a rigid lever. In this way, the “peri-talar and subtalar” structures are responsible for absorbing energy during the first contact of the foot with the ground, and then for transferring the centre of gravity from the back to the front and creating the rigid lever needed for the push-off [8]. For this reason, in people with excessive valgus heel, the foot does not fulfil the function of a rigid lever, thus enabling dynamic knockout after rolling the foot, and the short foot muscles and calf muscles are required perform greater work to compensate. Cases of flatfoot require increased work by the peroneus longus, tibialis posterior and anterior muscles, which may explain the calf cramps associated with this condition, as well as a feeling of heaviness or fatigue after prolonged walking [29].

Kinematic examinations did not reveal any major deviations between symptomatic and asymptomatic flat-valgus feet, the only difference found to date was medial displacement of the talus relative to the scaphoid, as assessed by x-ray [8, 30]. This however, requires further investigation. Based on these premises, it can be assumed that the correction of excessive valgus of the tarsus and reduction of talus dislocation found after arthroeresis may improve the biomechanics of foot rolling and make the symptomatic foot painless.

The main goal of treating flatfoot should be the elimination of pain; however, this does not necessarily entail the elevation of the longitudinal arch; failing to do so may impair the function of the foot in the long run, as emphasized by Moraleda and Mubarak [8]. Even so, although not all patients achieved full correction of deformity, as indicated by the normalization of the measured parameters, arthroeresis should nevertheless be considered as an option where conservative treatment with insoles is ineffective: the procedure is characterized by an acceptable incidence of complications and high patient satisfaction. Particularly good results are obtained in patients with a subtalar-type deformity, which should be an important criterion for qualification for surgery. In other cases, it may be justified to supplement the operation with additional procedures or to choose a different operational technique.

It is worth mentioning, that some researchers describe more conservative approaches to the treatment of paediatric flatfoot. Martínez-Nova at al. report that among 1032 children, foot posture tended to shift toward neutral with age: within three years, based on foot posture index, the number of supinated and neutral feet increased significantly over time, while that of pronated feet decreased [2]. Another paper by the same authors included similar findings, and confirmed the lack of any clear relationship between higher BMI and flatfeet [31]. A review by Evans et al. found no evidence supporting the use of foot orthoses for treating healthy children with flexible flat feet. However, attention should be paid to paediatric foot conditions which cause pain, limit function, or reduce quality of life, such as cerebral palsy or juvenile idiopathic arthritis [7].

A limitation of the present study is that it is based on a relatively small number of patients; however, the numbers were sufficient for the statistical analysis, and for reliable conclusions to be drawn. In addition, the study design did not include a control group, and the follow-up was relatively short. Further research with longer follow-ups is needed to confirm whether the observed improvements in pain and function are durable, and whether they are due to the surgery or a natural improvement with time.

To conclude, subtalar arthroereisis is a minimally-invasive and effective method of surgical treatment for symptomatic, idiopathic, flexible flatfoot. It is characterised by an acceptable complication rate and good early clinical results.

## Data Availability

The dataset generated during and analyzed during the current study are not publicly available. An anonymized version of the dataset is available from first author of the study (A.S.) on reasonable request.
